# Serum Type 2 Cytokine Levels Are Elevated in a Chronic Pulmonary Aspergillosis Subgroup with High Serum Total Immunoglobulin E Level

**DOI:** 10.3390/jof11040303

**Published:** 2025-04-10

**Authors:** Shizuka Watanabe, Junko Suzuki, Maho Suzukawa, Taku Nishimura, Masato Watanabe, Yu Enomoto, Keita Takeda, Kei Kusaka, Masahiro Kawashima, Yoshiteru Morio, Atsuhisa Tamura, Hideaki Nagai, Yuka Sasaki, Hirotoshi Matsui

**Affiliations:** 1 Clinical Research Center, National Hospital Organization Tokyo National Hospital, Tokyo 204-8585, Japan; fueta-tky@umin.ac.jp (M.S.);; 2 Department of Respiratory Medicine, University of Tokyo, Tokyo 113-8654, Japan; 3 Center for Pulmonary Diseases, National Hospital Organization Tokyo National Hospital, Tokyo 204-8585, Japankawashima.masahiro.wr@mail.hosp.go.jp (M.K.); morio.yoshiteru.pn@mail.hosp.go.jp (Y.M.); tamura.atsuhisa.py@mail.hosp.go.jp (A.T.); hnagai-in@outlook.jp (H.N.); sasaki.yuka.vh@mail.hosp.go.jp (Y.S.);; 4 Asthma, Allergy and Rheumatology Center, National Hospital Organization Tokyo National Hospital, Tokyo 204-8585, Japan

**Keywords:** chronic pulmonary aspergillosis, serum total IgE, cytokine, pulmonology, Th2 inflammation

## Abstract

A subgroup of patients with chronic pulmonary aspergillosis (CPA) exhibits elevated serum total immunoglobulin E (IgE) levels, similar to allergic bronchopulmonary aspergillosis; however, the underlying mechanisms remain unclear. This study aimed to clarify the underlying pathophysiology of the CPA subgroup with high serum total IgE levels. In this study, we prospectively collected CPA cases treated at our hospital between January and July 2022 and measured serum cytokine levels along with clinical data. We compared 34 healthy controls (HCs) and 51 patients with CPA and found significantly elevated levels of inflammatory cytokines and tissue repair and destruction-related cytokines in CPA. Among the 51 patients with CPA, 10 had total IgE levels of >500 IU/mL, whereas the remaining 41 did not. The IgE-high group exhibited significantly increased eosinophil counts and elevated levels of type 2 cytokines and pro-inflammatory cytokines. Based on these findings, patients with CPA exhibited an enhanced inflammatory response in terms of cytokines compared with HCs. In particular, the CPA subgroup with high total IgE levels may have an underlying enhancement of type 2 inflammation. Our study provides insights into the potential novel pathomechanisms of CPA and may contribute to the development of new treatment strategies.

## 1. Introduction

Aspergillus can cause various diseases in humans. Notable examples include allergic bronchopulmonary aspergillosis (ABPA), which results from a type 2 immune response to Aspergillus in patients with asthma or cystic fibrosis; chronic pulmonary aspergillosis (CPA), which occurs in patients with normal or mildly compromised immunity and pre-existing lung structural damage; and invasive pulmonary aspergillosis, which affects immunocompromised patients [1]. The 5-year survival rate of CPA is approximately 65% [2,3], highlighting the urgent need for a better understanding of its pathophysiology and the development of novel treatment strategies.

Sehgal et al. reported that among 269 patients with CPA without asthma or a history of treatment, 34.6% had serum total immunoglobulin E (IgE) > 500 IU/mL, and 11.2% had blood eosinophil counts (BECs) ≥ 500/μL, suggesting the presence of a subgroup with a blood immunological profile similar to ABPA [4,5]. Additionally, we previously reported that in patients with CPA and high serum total IgE levels, total IgE levels fluctuated with disease progression and remission, indicating its potential as a biomarker, similar to that in ABPA [6]. Immunohistochemical analysis of resected lung specimens revealed mast cell infiltration in CPA lesions, regardless of serum total IgE levels. However, in CPA cases with high serum total IgE levels, IgE expression was notably elevated in the lesions, and co-staining of mast cells and IgE was observed [6]. Mast cells are involved in fungal defense [7,8], but in ABPA, antigen–IgE crosslinking activates mast cells, leading to the release of chemical mediators that recruit eosinophils and exacerbate inflammation [9]. These findings suggest that type 2 immune responses may contribute to the pathophysiology of this CPA subgroup.

This study aimed to clarify the underlying pathophysiology of the CPA subgroup with high serum total IgE levels based on the hypothesis that the disease mechanism differs from that of other CPA cases. Although previous studies have investigated cytokine levels in patients with CPA, the sample size was small [10,11]. One study that compared CPA and ABPA reported lower levels of type 2 helper T (Th2) cytokines, such as interleukin (IL)-5 and IL-13, in CPA [10]. However, that study included only 10 CPA cases, with a mean serum total IgE level of 105.1 ± 184.8 IU/mL (normal range, <170 IU/mL), which does not sufficiently represent the subgroup of interest. Therefore, we first compared cytokine levels between healthy controls (HCs) and patients with CPA and subsequently analyzed cytokine profiles within CPA by comparing those with high serum total IgE levels to other CPA cases. The presence of enhanced type 2 inflammation in this CPA subgroup may provide new insights into its pathophysiology and lead to the development of novel therapeutic approaches.

## 2. Materials and Methods

### 2.1. Patient Characteristics

We identified patients with CPA from a list of patients at the National Hospital Organization Tokyo National Hospital who used voriconazole or itraconazole between January and July 2022. The CPA diagnostic criteria were based on the guidelines of the Infectious Diseases Society of America (IDSA) [12]. They included positive anti-*Aspergillus* antibodies in the serum or a bacteriological/pathological proof of *Aspergillus* in respiratory organ specimens, the lack of or mild immune deficiency, and the presence of underlying lung disease, in addition to symptoms persisting ≥ 3 months and characteristic imaging findings. All imaging findings were confirmed by at least two experienced pulmonologists. Patients with fungal diseases other than CPA, such as ABPA, and those diagnosed with CPA who did not meet the confirmed diagnostic criteria after 2014 were excluded. To eliminate the effects of cytokines, patients undergoing treatment for non-tuberculous mycobacterial lung disease (NTM), tuberculosis, or malignancies, those with concurrent interstitial lung diseases identifiable through chest radiography or collagen diseases, and those on steroids or with immune deficiencies, such as human immunodeficiency virus/acquired immunodeficiency syndrome (HIV/AIDS), were excluded. Patients who underwent CPA surgery and showed no lesions on CT scans and those within 3 months post-CPA surgery were also excluded. HCs, similar to patients with CPA, were staff members who had not undergone treatment for NTM or other diseases. The study protocol was approved by the Ethics Committee of Tokyo National Hospital (approval no. 220075, 6 February 2025), and written informed consent was obtained from all the patients and HCs.

### 2.2. Data and Sample Collection

Serum samples from patients with CPA were stored in residual blood samples collected during outpatient visits for blood tests. Data collected included age, sex, age at diagnosis, disease duration, comorbidities (asthma, atopic dermatitis, diabetes, old pulmonary tuberculosis, chronic respiratory diseases), smoking status, and results from blood tests (serum total IgE levels, BECs, C-reactive protein (CRP), and *Aspergillus*-specific IgE), bacterial and pathological examinations, imaging studies, and whether bronchial artery embolization was performed, all gathered from medical records or questionnaires with attending physicians. Imaging findings were confirmed by two or more pulmonologists specializing in CPA. The HCs were staff members. Residual serum from blood samples collected during health checkups was used in this study. Information regarding age, sex, comorbidities (atopic dermatitis, diabetes, old pulmonary tuberculosis, chronic respiratory diseases), and smoking status was collected using a questionnaire. To elucidate the pathogenesis of the CPA subgroup with elevated serum total IgE levels, the patients were divided into two groups, those with serum total IgE levels < 500 IU/mL and those with serum total IgE levels > 500 IU/mL, based on previous studies [4,6] and the diagnostic criteria for ABPA by ISHAM and Asano et al. [13,14]. The serum was separated by centrifuging the blood samples and stored at −20 °C until cytokine levels were measured.

### 2.3. Serum Cytokine Levels

Serum samples were used to measure the levels of the following 32 cytokines using Human Bio-Plex Pro (BioRad Laboratories; Hercules, CA, USA) and LUMINEX 200 (Luminex; Austin, TX, USA) according to the manufacturers’ instructions: interferon (IFN)-γ; IL-1Ra, IL-2, IL-4, IL-5, IL-6, IL-7, IL-12p70, and IL-13; IL-18/IL-1F4; IL-25; IL-33; C-C motif chemokine ligand (CCL) 2 (CCL2)/monocyte chemoattractant protein (MCP)-1; CCL3/macrophage inflammatory protein (MIP)-1α; CCL4/MIP-1β; CCL5/regulated on activation normal T cell expressed and secreted (RANTES); CCL11/eotaxin; CCL17/thymus and activation-regulated chemokine (TARC); CXCL8/IL-8; CXCL10/IFN-inducible protein-10; chitinase-3-like protein 1 (CHI3-L1); leptin; matrix metalloproteinase (MMP)-1, MMP-3, MMP-8, and MMP-12; platelet-derived growth factor-BB (PDGF-BB); periostin; ST2/IL-33R; transforming growth factor (TGF)-β1; tissue inhibitor MMP-1; and tumor necrosis factor (TNF)-α.

*Aspergillus* immunoglobulin G (IgG) antibodies were measured by an enzyme-linked immunosorbent assay using Platelia *Aspergillus* IgG (Bio-Rad Laboratories, Hercules, CA, USA). Total IgE and *Aspergillus*-specific IgE were measured using the fluorescence enzyme immunoassay method.

### 2.4. Statistical Analyses

Statistical analyses were performed using a two-tailed *t*-test or one-way analysis of variance. All data are presented as means. Statistical analyses were performed using the GraphPad Prism 9 software version 9.5.1 (GraphPad Software, San Diego, CA, USA). A *p*-value < 0.05 was defined as statistically significant.

## 3. Results

### 3.1. Patient Characteristics (HCs vs. Patients with CPA)

**Figure 1:**
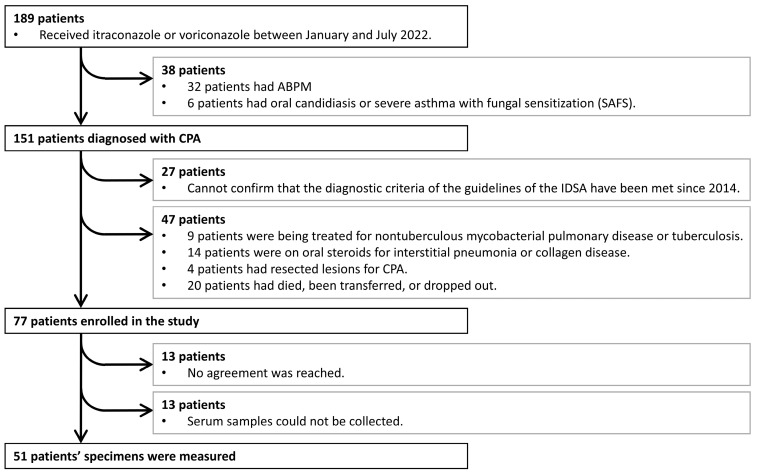
Flowchart of patient enrollment. ABPM, allergic bronchopulmonary mycosis; CPA, chronic pulmonary aspergillosis; IDSA, Infectious Diseases Society of America.

During the study period, 189 patients were treated with antifungal agents, 151 of whom were diagnosed with CPA (Figure 1). After excluding patients who did not meet the IDSA diagnostic criteria confirmed after 2014, those with comorbidities, such as NTM or corticosteroid use, those with unidentifiable lesion sites, and those who died or dropped out, 77 patients were included in this study. Ultimately, 51 patients provided informed consent, and cytokine measurements and other analyses were performed on their samples.

The background characteristics of HCs and patients with CPA are shown in Table 1. Compared with the HC group, the CPA group had a higher median (quartile) age of 55 (42.5–53.0) years, a greater proportion of males (76.5%), and a higher smoking rate (52.9%). In addition, 14.7% of the HCs had allergic rhinitis. In the CPA group, background pulmonary diseases were more common, with chronic obstructive pulmonary disease (COPD) and old pulmonary tuberculosis found in 25.5% and 47.1% of the patients, respectively. The median number of exacerbations per year was 0.35 ± 0.63, the median CPA disease duration was 5 (1–10) years, the number of cavities was 1.45 ± 1.02, and 27.5% of the patients had cavity wall thickening. All patients received antifungal treatment for at least 1 month.

### 3.2. Cytokines (HCs vs. CPA)

**Figure 2:**
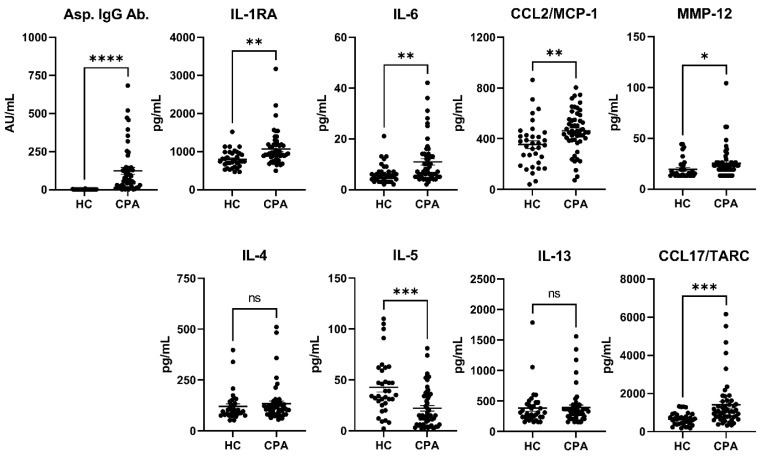
Cytokine levels (HCs vs. patients with CPA). HC vs. CPA. * *p* < 0.05, ** *p* < 0.01, *** *p* < 0.001, **** *p* < 0.0001, ns: not significant. Values are presented as the means ± standard errors of the mean.

In the CPA group, the median (IQR) and range of total IgE were 83.0 (26.0–304.0) and 25–5502 IU/mL, and those of *Aspergillus*-specific IgE were 0.25 (0.10–4.90) and 0.10–29.70 UA/mL. Compared with the HCs, patients with CPA had significantly higher levels of *Aspergillus* IgG antibodies (Table 2, Figure 2). The levels of inflammatory cytokines and chemokines, such as IL-1RA, IL-6, IL-18, and CCL2/MCP-1, and tissue remodeling-related markers, such as CHI3-L1 (*p* = 0.0003), MMP-8 (*p* = 0.013), and MMP-12 (*p* = 0.049), were significantly elevated. The levels of the Th2 inflammation marker CCL17/TARC (*p* = 0.0009) and the receptor for IL-33, ST2/IL-33R (*p* = 0.0006), which are secreted by group 2 innate lymphoid cells, were also elevated. Conversely, the levels of leptin (*p* = 0.042), TGF-β1 (*p* < 0.0001), and IL-5 (*p* = 0.0001), which mobilize eosinophils, were significantly lower in the CPA group than in the HC group.

### 3.3. Patient Characteristics (IgE Levels < or ≥500 IU/mL in Patients with CPA)

Next, we compared cytokines and other markers between patients with CPA with and without elevated serum total IgE levels. The patient background data are shown in Table 3. The group with total IgE levels < 500 IU/mL included 41 patients, whereas the group with total IgE levels > 500 IU/mL included 10 patients. No significant differences were found between the groups in terms of sex, allergic diseases, underlying pulmonary diseases, number of cavities, or the presence of cavity wall thickening.

### 3.4. Cytokines (Total IgE Levels < or ≥500 IU/mL in Patients with CPA)

**Figure 3:**
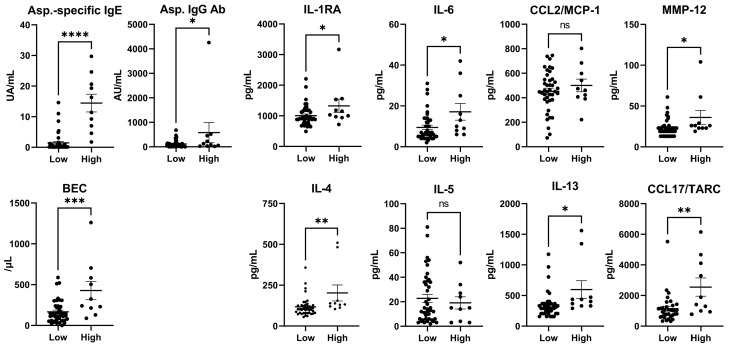
Cytokine levels (total IgE levels < or ≥500 IU/mL in patients with CPA). Total IgE levels < 500 IU/mL (low) vs. total IgE ≥ 500 IU/mL (high) in patients with CPA. * *p* < 0.05, ** *p* < 0.01, *** *p* < 0.001, **** *p* < 0.0001, ns: not significant. Values are presented as the means ± standard errors of the mean.

In the group with serum total IgE levels > 500 IU/mL, *Aspergillus*-specific IgE, *Aspergillus* IgG antibody, and BEC were significantly higher compared with the group with lower serum total IgE levels (Table 4, Figure 3). The levels of cytokines and chemokines associated with Th2 inflammation, such as IL-4 (*p* = 0.0062), IL-13 (*p* = 0.011), IL-25 (*p* = 0.006), and CCL17/TARC (*p* = 0.0021), were significantly elevated. Additionally, inflammatory cytokines and chemokines, including IL-1RA, IL-2, IL-6, IL-7, IL-12p70, TGF-β1, TNF-α, CCL3/MIP-1α, and CCL4/MIP-1β, and remodeling-related markers, such as MMP-1, MMP-12, PDGF-BB, and TIMP-1, were also significantly elevated.

### 3.5. Case Presentation

**Figure 4:**
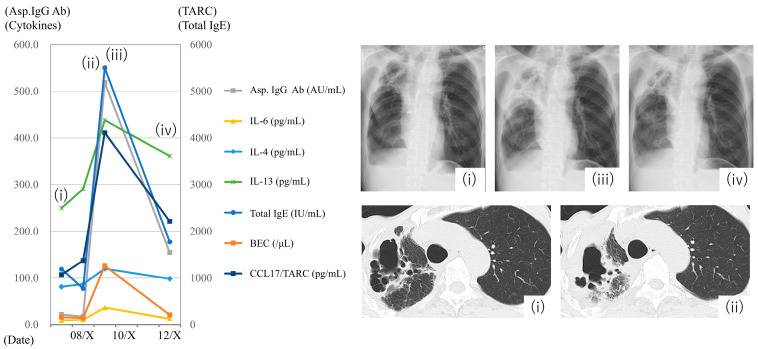
Case presentation.

Data from a patient with CPA in his 60s are shown in chronological sequence (Figure 4). The patient had a history of diabetes, COPD, and lung cancer (treated with surgery). In September of year X-1, the patient was diagnosed with CPA, and itraconazole treatment was initiated. By March X, the patient’s condition had stabilized, and discontinuation of antifungal therapy was considered (Figure 4, chest radiography and computed tomography [CT] (i)). However, in early August of year X, the patient developed a fever, and penicillin-based antibiotics showed partial efficacy. In late August, the inflammatory markers and pericavitary shadows in the right upper lobe worsened, leading to a diagnosis of CPA exacerbation (Figure 4, chest radiography and CT (ii–iii)). Treatment with micafungin and voriconazole improved symptoms, inflammatory markers, and imaging findings, and the patient was discharged while continuing voriconazole treatment. By December X, the patient remained stable (Figure 4, chest radiography and CT (iv)).

Changes in BEC, serum total IgE, *Aspergillus*-specific antibodies, and various cytokines before and after the CPA exacerbation are shown in Figure 4. During the exacerbation in late August of year X, both *Aspergillus* IgG and serum total IgE levels increased, indicating an increase in the *Aspergillus* bacterial population. Simultaneously, the levels of inflammatory cytokines, such as IL-1RA and IL-6; markers of type 2 inflammation, such as BEC, IL-4, IL-13, and CCL17/TARC; and remodeling-related markers, such as MMP, also increased. The expression of these markers decreased as the CPA condition improved.

CPA in a patient in his/her 60s with a history of diabetes, chronic obstructive pulmonary disease, and lung cancer surgery. The patient was diagnosed with CPA in September of year X-1 and started treatment with itraconazole. By March X, the patient’s condition had stabilized. In early August of year X, the patient developed a fever, and CPA exacerbation was later diagnosed. The antifungal treatment was switched to voriconazole, after which the patient’s condition stabilized. The left panel illustrates changes in blood eosinophil count (BEC), total immunoglobulin E (IgE), *Aspergillus* IgG antibody levels, and various cytokines before and after CPA exacerbation in August of year X. The right panels display radiographs and CT images corresponding to the time points (i)–(iv) in the graph: (i) March of year X, (ii) late August, (iii) early September, and (iv) December.

## 4. Discussion

To date, a subgroup of CPA characterized by elevated serum total IgE levels has been identified [4,5], and serum total IgE has been suggested to correlate with disease activity [6]. By measuring cytokines, this study confirmed that this subgroup not only exhibited elevated levels of inflammatory cytokines, such as IL-6, but also presented a profile indicative of type 2 inflammation, including eosinophilia, and increased levels of IL-4 and IL-13, suggesting the presence of type 2 inflammation similar to that observed in ABPA.

First, we compared the cytokine levels between HCs and patients with CPA (Table 1 and Table 2, Figure 2). Because residual serum from health checkups of hospital staff, including nurses, was used, the HC group was relatively younger, with a higher proportion of females and fewer smokers than the CPA group (Table 1). In patients with CPA, the levels of inflammatory cytokines and chemokines, such as IL-1RA, IL-6, IL-18, and CCL2/MCP-1, were significantly elevated [15,16,17]. Levels of markers related to tissue remodeling, including CHI3-L1, MMP-8, MMP-12, and TGF-β1, were also significantly increased, suggesting tissue destruction and repair in CPA [18,19]. Thus, cytokine data showed that inflammation and the resulting tissue destruction and repair were observed in patients with CPA compared with HCs. Additionally, leptin levels were significantly lower in the CPA group than in the HC group, which may be related to the low body weight characteristics of the CPA group, as leptin is secreted by adipocytes [12]. Significantly lower levels of IL-5, a potent eosinophil-activating factor, may reflect a negative feedback mechanism due to increased and activated eosinophils in the CPA group. Furthermore, IFN-γ showed signs of functional impairment in patients with CPA, and a randomized controlled trial is underway to assess the addition of IFN-γ to antifungal therapy [20,21] (clinicaltrials.gov; NCT05653193). However, no decrease in blood concentration of IFN-γ was observed.

Next, we divided the patients with CPA into two subgroups, those with a serum total IgE level of ≥500 IU/mL and those with lower levels, and we compared them. There were no significant differences in patient backgrounds (Table 3). The subgroup with total IgE ≥ 500 IU/mL consisted of 10 (19.6%) patients, which was lower than that in previous studies that included only untreated patients with CPA [4,5]. However, as many of the patients in our study were already undergoing treatment, and total IgE levels may decrease as disease activity is controlled [6], this lower proportion in our subgroup can be reasonably explained.

In the subgroup with elevated serum total IgE levels, in addition to the increased inflammatory cytokine and MMP characteristic of CPA, we observed a significant increase in the levels of BECs and type 2 cytokines, such as IL-4, IL-13, and CCL17/TARC (Table 4, Figure 3). In our previous study, we demonstrated that CPA lesions in patients with elevated serum total IgE levels contained a large number of mast cells co-stained with IgE [6]. Mast cells act as sentinels of the immune system and express receptors, such as Toll-like receptors and C-type lectin receptors that recognize fungi on their surfaces [7,8]. They also secrete type 2 cytokines and chemokines, including IL-4, IL-13, and IL-25, and inflammatory cytokines, such as IL-6, TNF-α, CCL3/MIP-1α, and CCL4/MIP-1β, which were elevated in this study [22,23]. CCL17/TARC is a chemokine that primarily binds to CCR4 and is expressed in Th2 cells. It is associated with disease severity in conditions such as atopic dermatitis and asthma [24] and regulates CCL5-induced mast cell migration via CCR4 in vivo [25]. Taken together, these findings suggest that a subgroup of patients with CPA and high serum total IgE levels may have coexisting type 2 inflammation, similar to that observed in ABPA. A randomized clinical trial of corticosteroids, a commonly used treatment for ABPA [13], is currently underway for this subgroup (clinicaltrials.gov, NCT05444946). Furthermore, our results suggest that biologics, such as anti-IgE antibodies, which have shown efficacy in ABPA and have fewer side effects, including a lower risk of infections than corticosteroids [26], may also be beneficial for this subgroup. In addition, levels of IL-5, a potent eosinophil chemoattractant, and activator, did not differ significantly in this study. Previous reports on cases of eosinophilic asthma successfully treated with anti-IL-5 or anti-IL-5 receptor antibodies also found no increase in serum IL-5 levels [27,28,29], suggesting a possible discrepancy between tissue and serum concentrations. Additionally, the higher *Aspergillus*-specific IgE and *Aspergillus*-IgG antibody levels in the high serum total IgE subgroup compared with the low serum total IgE group suggests a possible association with fungal load [30]. This is supported by the presence of subgroups in which serum total IgE levels increased during CPA exacerbation [6]. Furthermore, in cases wherein we could track the disease course, including periods of CPA exacerbation, we measured cytokine levels at multiple time points (Figure 4). We observed that the total levels of IgE, BECs, *Aspergillus*-specific IgG antibodies, inflammatory cytokines, and type 2 cytokines fluctuated in parallel with disease activity, supporting our findings.

This study has some limitations. First, this study was conducted at a single institution and was not a large-scale study. Second, we measured serum cytokine levels rather than cytokine concentrations at local lesion sites. Third, it is possible that some CPA cases in our study were complicated by coexisting ABPA. Among the 51 patients, four met all the essential components of the new ISHAM diagnostic criteria [13], namely, elevated *A. fumigatus*-specific IgE and elevated total serum IgE, and the other components, including elevated *A. fumigatus*-specific IgG level and blood eosinophilia. However, none of the patients had a history of asthma or cystic fibrosis. Additionally, these patients did not exhibit high-attenuation mucus or expectoration of mucus plugs, which are characteristic features of ABPA according to the new diagnostic criteria. Instead, they presented radiological findings that were more consistent with CPA, such as cavitation and pleural thickening [12]. Based on these observations, we concluded that these cases were unequivocal CPA rather than ABPA. Finally, as this was a cross-sectional study and all patients were already undergoing treatment for a minimum of 1 month, inflammatory markers and cytokine levels were likely lower than those observed in studies of treatment-naive patients. However, CPA is difficult to control, as evidenced by its low 5-year survival rate [2,3]. In a cross-sectional study that included treated patients, many patients exhibited persistent inflammation (Table 2). Therefore, we believe that this study provides sufficient information to elucidate the disease pathology. Additionally, the subgroup with serum total IgE levels within 500 IU/mL included cases in which the total IgE levels were initially >500 IU/mL at the start of treatment. As total IgE levels may fluctuate depending on the disease activity of CPA [6], further studies should be conducted by collecting more treatment-naive cases and investigating clinical characteristics, including the severity of inflammation and prognosis, in subgroups with high total IgE levels. Comparing cytokine dynamics before and after treatment and during remission and exacerbation can provide insights into which drugs targeting type 2 inflammation are the most effective and help determine the optimal timing for their administration.

## 5. Conclusions

Patients with CPA exhibit elevated levels of cytokines associated with inflammation and tissue destruction compared with HCs. In CPA, the presence of a subgroup characterized by elevated total serum IgE levels has drawn attention, suggesting a correlation between CPA disease activity and total serum IgE levels [6]. In this study, we found that this subgroup exhibited not only an increase in the levels of inflammatory cytokines, such as IL-6 but also a profile indicative of type 2 inflammation, including peripheral blood eosinophilia and elevated serum IL-4 and IL-13 levels. These findings suggest that this subgroup, despite having CPA, may simultaneously exhibit an immune response similar to that observed in ABPA. CPA is a challenging disease to treat; however, corticosteroids and biologics, such as anti-IgE antibodies, which are used in ABPA [13], may serve as potential new therapeutic options for this subgroup.

## Figures and Tables

**Table 1:** Patient characteristics (HCs vs. patients with CPA).

	HCs		Patients with CPA		*p*-Value	
	n = 34		n = 51			
Age (years)	42.5	(35.0–53.0)	69.0	(55.0–77.0)	<0.0001	****
Men	11	32.4%	39	76.5%	<0.0001	****
Smoking	8	23.5%	27	52.9%	0.008	**
Bronchial asthma	6	17.6%	6	11.8%	0.53	ns
Atopic dermatitis	3	8.8%	1	2.0%	0.29	ns
Allergic rhinitis	5	14.7%	1	2.0%	0.035	*
Diabetes mellitus	-		7	13.7%	-	
Old tuberculosis	-		24	47.1%	-	
NTM	-		9	17.6%	-	
COPD	-		13	25.5%	-	
Number of exacerbations per year	-		0.35	±0.63	-	
Number of cavities	-		1.45	±1.02	-	
Wall thickness < 3 mm	-		14	27.5%	-	
BAE	-		12	23.5%	-	

Age and duration of morbidity are shown as medians (quartiles). Number of exacerbations per year and the number of cavities are shown as means ± standard deviations. * *p* < 0.05; ** *p* < 0.01, **** *p* < 0.0001. HCs, healthy controls; CPA, chronic pulmonary aspergillosis; ns: not significant; NTM, nontuberculous mycobacterial infection; COPD, chronic obstructive pulmonary disease; BAE, bronchial arterial embolization.

**Table 2:** Cytokine levels (HCs vs. patients with CPA).

	HCs	Patients with CPA	*p*-Value	
Total IgE (IU/mL)	-	345.7 ± 827.6	-	
Asp.-specific IgE (UA/mL)	-	4.0 ± 7.1	-	
CRP (mg/dL)	-	0.9 ± 1.8	-	
BEC (/μL)	-	218.1 ± 221.5	-	
Albumin (g/dL)	-	3.9 ± 0.5	-	
HbA1c (%)	-	6.2 ± 1.0	-	
Asp. IgG Ab. (AU/mL)	0.4 ± 1.1	205.3 ± 598.5	<0.0001	****
IFN-γ	79.6 ± 155	69.4 ± 156.9	0.77	
IL-1RA	794 ± 230.3	1067 ± 443.7	0.0014	**
IL-2	17.5 ± 14.1	16.7 ± 11.4	0.78	
IL-4	120.4 ± 72.4	133.7 ± 90.7	0.48	
IL-5	42.7 ± 27.4	22 ± 19.7	0.0001	***
IL-6	6.2 ± 3.9	11 ± 8.8	0.0041	**
IL-7	35.8 ± 18.2	38.6 ± 15.6	0.46	
IL-12p70	161.2 ± 164.1	185.2 ± 188.7	0.55	
IL-13	384.9 ± 306.7	395.2 ± 287.1	0.88	
IL-18	173.8 ± 63.3	288.1 ± 283.5	0.024	*
IL-25	272.3 ± 232.8	269 ± 168.3	0.94	
IL-33	31.8 ± 45.9	33.4 ± 75.1	0.91	
CCL2/MCP-1	353 ± 180.3	461.9 ± 162.9	0.0049	**
CCL3/MIP-1α	315.4 ± 77.2	341.7 ± 73.9	0.12	
CCL4/MIP-1β	583.4 ± 167.3	627.3 ± 190.8	0.28	
CCL5/RANTES	30,349 ± 14,838	30,039 ± 15,215	0.93	
CCL11/eotaxin	215.7 ± 135.2	271 ± 262.5	0.26	
CCL17/TARC	651.1 ± 330.2	1410 ± 1247	0.0009	***
CXCL8/IL-8	18.1 ± 10.2	21.7 ± 10.6	0.12	
CXCL10/IP-10	12.9 ± 3.8	15 ± 9.3	0.21	
CHI3-L1	41,817 ± 53,524	202,221 ± 242,726	0.0003	***
Leptin	17,925 ± 18,791	10,720 ± 13,404	0.042	*
MMP-1	3399 ± 2412	4146 ± 2122	0.14	
MMP-3	18,597 ± 8392	24,988 ± 18,494	0.062	
MMP-8	821.6 ± 430.2	1536 ± 1609	0.013	*
MMP-12	19.5 ± 9.5	25.5 ± 15.5	0.049	*
PDGF-BB	7617 ± 2903	8443 ± 2837	0.20	
Periostin	250,336 ± 86,290	241,604 ± 94,467	0.67	
ST2/IL-33R	22,872 ± 6835	29,079 ± 8477	0.0006	***
TGF-β1	25,185 ± 9017	17,296 ± 6234	<0.0001	****
TIMP-1	130,314 ± 32,424	134,878 ± 25,525	0.47	
TNF-α	10.8 ± 7.8	10.6 ± 6.2	0.89	

Values are presented as the means ± standard deviations. The units for each cytokine are expressed in pg/mL. * *p* < 0.05; ** *p* < 0.01, *** *p* < 0.001, **** *p* < 0.0001. Asp., *Aspergillus*; IgE, immunoglobulin E; CRP, C-reactive protein; BEC, blood eosinophil count; HbA1c, hemoglobin A1c; IgG, immunoglobulin G; Ab, antibody; IFN, interferon; IL, interleukin; CCL, chemokine C-C motif ligand; MCP, monocyte chemotactic protein; MIP, macrophage inflammatory protein; RANTES, regulated on activation normal T cell expressed and secreted; TARC, thymus and activation-regulated chemokine; CXCL, C-X-C motif chemokine ligand; CHI3-L1, chitinase-3-like protein 1; MMP, matrix metalloproteinase; PDGF-BB, platelet-derived growth factor-BB; TGF, transforming growth factor; TIMP, tissue inhibitor matrix metalloproteinase; TNF, tumor necrosis factor.

**Table 3:** Patient characteristics (total IgE levels < or ≥500 IU/mL in patients with CPA).

	Total IgE < 500 IU/mL		Total IgE ≥ 500 IU/mL		*p*-Value	
	n = 41		n = 10			
Age (years)	69	(57.5–77.0)	55.5	(49.0–72.0)	0.11	ns
Men	31	75.6%	8	80.0%	>0.99	ns
Smoking	20	48.8%	7	70.0%	0.30	ns
Bronchial asthma	3	7.3%	3	30.0%	0.08	ns
Atopic dermatitis	0	0%	1	10.0%	0.20	ns
Allergic rhinitis	1	2.4%	0	0.0%	0.20	ns
Diabetes mellitus	4	9.8%	3	30.0%	0.12	ns
Old tuberculosis	20	48.8%	4	40.0%	0.73	ns
NTM	7	17.1%	2	20.0%	>0.99	ns
COPD	11	26.8%	2	20.0%	>0.99	ns
Period of morbidity	5.0	(1.0–10.5)	3.5	(1.8–10.3)	0.90	ns
Number of exacerbations per year	0.29	±0.61	0.60	±0.70	0.17	ns
Number of cavities	1.3	±0.9	2.0	±1.33	0.058	ns
Wall thickness < 3 mm	12	29.2%	3	30.0%	>0.99	ns
BAE	8	19.5%	4	40.0%	0.22	ns

Age and duration of morbidity are shown as medians (quartiles). Number of exacerbations per year and the number of cavities are shown as means ± standard deviations. ns: not significant.

**Table 4:** Cytokine levels (total IgE levels < or ≥500 IU/mL in patients with CPA).

	Total IgE < 500 IU/mL	Total IgE ≥ 500 IU/mL	*p*-Value	
Total IgE (IU/mL)	95.8 ± 103.8	1370.0 ± 1519.0	<0.0001	****
Asp.-specific IgE (UA/mL)	1.4 ± 3.1	14.5 ± 9.1	<0.0001	****
CRP (mg/dL)	0.8 ± 1.6	1.5 ± 2.2	0.24	
BEC (/μL)	166.9 ± 139.0	427.6 ± 355.0	0.0005	***
Albumin (g/dL)	3.9 ± 0.5	3.7 ± 0.6	0.24	
HbA1c (%)	6.0 ± 0.6	6.7 ± 1.6	0.084	
Asp. IgG Ab. (AU/mL)	113.9 ± 145.9	580.1 ± 1304.0	0.026	*
IFN-γ	55.3 ± 124.9	127.1 ± 250.6	0.2	
IL-1RA	1004.0 ± 340.3	1326.0 ± 697.4	0.038	*
IL-2	14.8 ± 7.0	24.5 ± 20.4	0.014	*
IL-4	116.9 ± 57.9	202.5 ± 155.7	0.0062	**
IL-5	22.8 ± 20.7	19.1 ± 15.6	0.6	
IL-6	9.5 ± 6.9	17.1 ± 12.9	0.013	*
IL-7	35.6 ± 11.9	50.9 ± 22.7	0.0044	**
IL-12p70	151.8 ± 116.3	322.1 ± 334.4	0.0091	**
IL-13	345.8 ± 207.9	597.5 ± 457.2	0.011	*
IL-18	302.9 ± 307.9	227.3 ± 141.8	0.46	
IL-25	237.7 ± 123.6	397.1 ± 258.3	0.006	**
IL-33	28.4 ± 67.1	53.5 ± 103.9	0.35	
CCL2/MCP-1	452.5 ± 163.1	500.4 ± 164.5	0.41	
CCL3/MIP-1α	325.6 ± 59.7	407.6 ± 92.2	0.0011	**
CCL4/MIP-1β	596.8 ± 162.9	752.6 ± 250.1	0.019	*
CCL5/RANTES	28,019 ± 12,809	38,319 ± 21,499	0.054	
CCL11/eotaxin	233.5 ± 127.1	424.4 ± 527.7	0.057	
CCL17/TARC	1131.0 ± 859.4	2553.0 ± 1883.0	0.0021	**
CXCL8/IL-8	21.5 ± 10.9	22.6 ± 9.5	0.77	
CXCL10/IP-10	15.0 ± 10.2	15.4 ± 4.9	0.89	
CHI3-L1	206,442 ± 223,098	184,915 ± 325,093	0.80	
Leptin	11,732 ± 14,368	6572 ± 7546	0.28	
MMP-1	3844 ± 2088	5385 ± 1877	0.038	*
MMP-3	24,600 ± 18,768	26,580 ± 18,194	0.76	
MMP-8	1445 ± 1634	1909 ± 1523	0.42	
MMP-12	22.9 ± 10.4	36.0 ± 26.7	0.012	*
PDGF-BB	7807 ± 1698	11,051 ± 4744	0.0007	***
Periostin	243,088 ± 87,985	235,522 ± 122,964	0.82	
ST2/IL-33R	28,433 ± 8605	31,729 ± 7774	0.27	
TGF-β1	16,432 ± 5450	20,840 ± 8158	0.044	*
TIMP-1	131,387 ± 24,488	149,191 ± 25,908	0.0047	*
TNF-α	9.4 ± 4.3	15.3 ± 10.0	0.0055	**

Values are presented as the means ± standard deviations. The units for each cytokine are expressed in pg/mL. * *p* < 0.05, ** *p* < 0.01, *** *p* < 0.001, **** *p* < 0.0001.

## Data Availability

The original contributions presented in this study are included in the article. Further inquiries can be directed to the corresponding author.

## Abbreviations

The following abbreviations are used in this manuscript:

ABPA	allergic bronchopulmonary aspergillosis
BECs	blood eosinophil counts
CCL	chemokine C-C motif ligand
CHI3-L1	chitinase-3-like protein 1
COPD	chronic obstructive pulmonary disease
CPA	chronic pulmonary aspergillosis
CRP	C-reactive protein
HCs	healthy controls
IDSA	Infectious Diseases Society of America
IFN	interferon
IgE	immunoglobulin E
IgG	immunoglobulin G
IL	interleukin
MCP	monocyte chemoattractant protein
MIP	macrophage inflammatory protein
MMP	matrix metalloproteinase
NTM	nontuberculous mycobacterial lung disease
PDGF-BB	platelet-derived growth factor-BB
RANTES	regulated on activation normal T cell expressed and secreted
TARC	thymus and activation-regulated chemokine
TGF	transforming growth factor
Th2	type 2 helper T
TNF	tumor necrosis factor
